# Censorship to an Extreme

**Published:** 1913-12-20

**Authors:** 


### CENSORSHIP TO AN EXTREME.

In the current number of the *English Review* there appears an article called "The Taboos of the British Museum," which points out crisply the disadvantages attaching to the practice of the authorities of the great Reading Room of that institution when dealing with works the subjects or the methods of which unfit them for popular perusal, such as those on sex matters or containing free criticism of contemporary European dynasties or of the Christian religion. With books of the two last descriptions we have naturally no concern here, but we are disposed to agree, in the main, with the author when he makes strictures upon the plan pursued with regard to the first-named kind. That plan is to take in the works and store them, but to mention their titles neither in the subject nor the author index and to give no information as to how they can be seen; in short, practically to bury them. Now, setting aside general arguments against this practice, which Mr. Haynes has already gone over, such as the facts that "readers" at the British Museum are adult persons vouched for by householders, and nearly all serious, steady devotees of some branch of learning, let us consider the particular disadvantages involved for medical men, certain of whom, such as alienists and neurologists (and ere very long there will probably be an English equivalent to the German *Sexualarzt*), must needs keep abreast of this literature. In London there are three big medical libraries: first, that of the College of Surgeons, by a good deal the finest of its kind in the British Isles; secondly, the College of Physicians' collection; and, thirdly, that of the Royal Society of Medicine. Now each of the two latter is confessedly less comprehensive than the first. The library in Pall Mall practically devotes itself to medical works of antiquarian interest, to iconography and so forth; and of these we believe that not more than a dozen or so are bought yearly. For contemporary works it relies on presents from its fellows, members, and licentiates. The one in Wimpole Street is a growing collection, but it is yet very young. We come then to that in Lincoln's Inn Fields. Now, although this is a very well found library, such as would be something of a revelation to many northern graduates, yet it labours under disadvantages as compared with its great neighbour in Bloomsbury. To begin with, it depends upon its own funds, enjoying no State support—a matter of importance when the rapid growth of medical literature is considered. It must buy its books; while the British Museum has them presented. Next—*bis dat qui cito dat*—it must in general wait until its able librarian, Mr. Plarr, has made his selection and brought that selection before a committee; whereas the national book repository gets a copy sent immediately a book is published. As a result the Royal College of Surgeons library cannot make up to medical men for the deprivation they suffer because of the old-maidishness in vogue at the British Museum. Reasonable restrictions of any kind everyone would welcome; they should not pass official wit to devise. Special readers' tickets might be issued to those willing and able to satisfy the authorities of their high purpose. A chamber of horrors might even be established for pornography.

But the present plan is discreditably wasteful and obscurantist. When Mr. Haynes' article meets Continental, and even American, eyes, we venture to say the spectacle of British prudery, which is afraid to let scholars read Havelock Ellis' "Psychologgy of Sex" at the author's expense (for he offered, it seems, to present that perforce foreign published work on condition it were catalogued, only to be refused), will lower the national prestige more than a good many foot-races lost at Olympic Games. Indeed, the London Library stocks standard books like that just mentioned, and is not afraid to say so.

